# Age and Type of Delivery as Risk Indicators for Maternal Mortality

**DOI:** 10.1055/s-0043-1768456

**Published:** 2023-04-27

**Authors:** Isabella Mantovani Gomes Dias de Oliveira, Emílio Prado da Fonseca, Fabiana Mantovani Gomes França, Karine Laura Cortellazzi, Vanessa Pardi, Antonio Carlos Pereira, Elaine Pereira da Silva Tagliaferro

**Affiliations:** 1 Campinas Health Department, Campinas, SP, Brazil; 2 Unifenas University, Divinópolis, MG, Brazil; 3 Restorative Dentistry and Dental Materials Division, São Leopoldo Mandic, Campinas, SP, Brazil; 4 Department of Health Sciences and Pediatric Dentistry, Piracicaba Dental School, State University of Campinas, Piracicaba, Brazil; 5 Department of Foundational Sciences, School of Dental Medicine, East Carolina University, Greenville, NC, USA; 6 Department of Community Dentistry, School of Dentistry, São Paulo State University, Araraquara, SP, Brazil

**Keywords:** Maternal mortality, Observational study, Socioeconomic factors, Mortalidade materna, Estudo observacional, Fatores socioeconômicos

## Abstract

**Objective:** This study assessed maternal mortality (MM) and related factors in a large-sized municipality in the Southeastern region of Brazil (Campinas, São Paulo) during the period 2000-2015.

**Methods:** This study consisted of two phases: 1. An analytical nested case-control phase that assessed the impact of individual and contextual variables on MM; and 2. an ecological phase designed to contextualize maternal deaths by means of spatial analysis. The case group consisted of all maternal deaths (n = 87) and the control group consisted of 348 women who gave birth during the same period. Data analysis included descriptive statistics, association, and multiple logistic regression (MLR) tests at p < 0.05 as well as spatial analysis.

**Results:** Maternal Mortality Ratio was 37 deaths per 100.000 live births. Deaths were dispersed throughout the urban territory and no formation of cluster was observed. MLR showed that pregnant women aged ≥ 35 years old (OR = 2.63) or those with cesarean delivery (OR = 2.51) were more prone to maternal death.

**Conclusion:** Maternal deaths were distributed dispersedly among the different socioeconomic levels and more prone to occur among older women or those undergoing cesarean deliveries.

## Resumo

**Objetivo:** Esse estudo avaliou a mortalidade materna (MM) e fatores relacionados em um município de grande porte da região sudeste do Brasil (Campinas, São Paulo) no período de 2000-2015.

**Métodos:** Esse estudo consistiu de duas fases: 1. Uma fase analítica de caso-controle que avaliou o impacto de variáveis individuais e contextuais na MM; 2. Uma fase ecológica delineada para contextualizar as mortes maternas por meio de análise espacial. O grupo caso consistiu de 87 mortes maternas e o grupo controle de 348 mulheres que tiveram bebês durante o mesmo período. Os dados foram analisados por estatística descritiva, testes de associação e regressão logística múltipla (RLM) (p < 0,05) assim como análise espacial.

**Resultados:** A taxa de mortalidade materna foi de 37 mortes para cada 100.000 nascidos vivos. As mortes foram dispersas por todo o território urbano e não se observou formação de clusters. Na RLM observou-se que mulheres grávidas com idade ≥ 35 anos (OR = 2,63) ou aquelas que passaram por cesárea (OR = 2,51) foram mais propensas à morte materna.

**Conclusão:** As mortes maternas foram distribuídas dispersamente entre os diferentes níveis socioeconômicos e mais propensas a ocorrer entre mulheres ≥ 35 anos de idade ou que passaram por cesárea.

## Introduction

Maternal mortality (MM) represents social and economic status in a country. Reducing MM is one of the goals of the Sustainable Development Goals (SDG) 1 and eliminating avoidable MM must be achieved by 2030. 1 2 3

Despite the reduction of 38% in global Maternal Mortality Ratio (MMR) from 2000 (342 deaths per 100,000 livebirths) to 2017 (211 deaths per 100,000 livebirths) 1 several challenges have to be overcome, especially in low- and middle-income countries. In Brazil, the MMR in 2017 was 60/100,000. 4 The Brazilian Ministry of Health has proposed actions such as the National Policy for Comprehensive Healthcare for Women, 5 the National Agreement for the Reduction of Neonatal and Maternal Mortality 6 and the Stork Network 7 that aim to reduce maternal mortality by means of qualified and humanized care.

Many factors can affect MM such as healthcare access, healthcare quality during antenatal care, childbirth and puerperium care, quality of obstetric emergencies assistance, 8 age, 9 10 years of education, 9 antenatal appointments, 1 marital situation, 9 and socioeconomic factors. 11 12 It is also known that maternal mortality rate is associated with healthcare system factors and the disease burden in a country. 11

Public policies for reducing maternal morbimortality require that the government takes specific and systematic actions on reformulating these policies. 13

MM surveillance and the analysis of factors associated with adverse outcomes are key to subside political decision-making and to contribute to an efficient resource allocation for MM reduction.

This study aimed to assess maternal mortality and related factors in Campinas, São Paulo, a large-sized municipality of the Brazilian Southeast region between 2000-2015.

## Methods

This observational study was performed in a large-sized municipality, Campinas, located in the countryside of São Paulo State, Brazil. At the time of data collection (2000-2015), Campinas presented Municipal Human Development Index of 0.805 14 and a population of 1,135,623 inhabitants with 33% of women in reproductive age. 15 A total of 63 (sixty-three) health centers, acting on the Family Health Strategy model, worked with well-delimited territories, client subscriptions, and multi-professional teams providing primary health care and some medium complexity procedures. Specialized care was provided by more than 20 public reference units and by the urgency and emergency system. 16 There are seven maternity units in the city and all of them assist women living in Campinas. One maternity assists Unified Health System (SUS) patients, two assist SUS and health insurance patients and four assist only private patients.

Two phases composed the study design: an analytical nested case-control phase that assessed the impact of individual and contextual variables on maternal mortality during the period 2000-2015, and an ecological phase designed to contextualize maternal deaths by means of descriptive and spatial analysis.

For the nested case-control phase, the case group was comprised of all maternal deaths (n = 87) registered in the Mortality Information System and investigated by the Municipal Committee of Maternal Mortality Surveillance during the period 2000-2015. 15 The control group was comprised of 348 women who gave birth during the same period and were randomly selected from the Information System on Live Births. The number of controls was determined using a 4:1 ratio, as there is no statistical gain to justify higher ratios. 17 The final sample size (n = 435) provided a test power of 0.80 (β = 20), a significance level of 5% (α = 0.05) for a minimum detectable Odds Ratio of 2.0.

Collected data included clinical variables: antenatal care (at least one appointment), number of antenatal appointments, number of previous pregnancies, number of fetuses and type of birth; as well as sociodemographic variables: age (years), education (completed study years), marital status/situation and the contextual variable: socioeconomic level. The Municipal Health Department built three socioeconomic levels (Low, Middle, and High) for each area covered by the healthcare clinics using data from the Demographic Census of 2000 and considering the following variables: percentage of people responsible for the household having an income of 10 minimum wages or more, percentage of people earning less than 2 minimum wages, percentage of people responsible for households that has more than 10 years of education, and percentage of people that has less than 1 year of education. According to this criteria, all the areas covered by the healthcare clinics were divided in order to guarantee a third of the population in each of those three socioeconomic levels. 18 After descriptive analysis of the data, regression logistic models were estimated to analyze the individual associations of each independent variable with the outcome variable. The variables age, education, marital status, antenatal care, number of antenatal appointments, pregnancy, and type of delivery were dichotomized based on a previous study 17 The variables that presented a p value of p≤0.20 in the individual association analysis (crude) were tested in a multiple logistic regression model, with variables at p≤0.05 remaining in the final model when analyzed together. All analyses were performed in the SAS Program (SAS Institute Inc., Cary, NC, USA, Release 9.2, 2010).

Thematic maps were designed to georefence maternal deaths according to socioeconomic levels. Two information plans were used: “points” which consisted of data about household address of the maternal death cases that were after converted into geographical coordinates by BatchGeo program 19 ; and “polygons” which consisted of the municipality's urban area divided into health units' coverage areas, whose data were provided by the Municipal Health Department. Both information layers were merged in QGIS Desktop® computer program (version 2.8.1).

Maternal Mortality Ratio (total of maternal deaths/total of live births*100,000) was calculated for the municipality and for each socioeconomic level.

This study was performed in accordance with the ethical standards laid down in the 1964 Declaration of Helsinki and its later amendments and approved by the Research Ethics Committee of the Piracicaba Dental School, State University of Campinas (Protocol 130/2014).

## Results

Maternal deaths (n = 87) occurred among women 16 to 44 years old. Most of them studied 4 years or more (96%), 24.5% graduated from high school and 13% held a college degree ( Table 1 ). The majority was married (52%), had more than 4 antenatal appointments (83%), had already been pregnant (61%) and had a single pregnancy (95%). A total of 81% underwent cesarean delivery.

**Table 1:** Sociodemographic characterization of the cases of maternal deaths and controls

Sociodemographic characteristics	n(%)	
Age (years)	Cases (87)	Controls (348)
< 35	63 (72.4)	304 (87.4)
≥ 35	24 (27.6)	44 (12.6)
Education (years)		
< 4	2 (3.8)	9 (2.6)
≥ 4	51 (96.2)	338 (97.4)
Marital status		
Single / Divorced / Widow	41 (48.2)	143 (41.1)
Married	44 (51.8)	205 (58.9)
Socioeconomic		
Low	46 (53.5)	157 (45.1)
Medium	23 (26.7)	102 (29.3)
High	17 (19.8)	89 (25.6)

Missing information: Education (n = 35), Marital status (n = 2), Socioeconomic (n = 1).

Among maternal deaths assessed by the Municipal Committee of Maternal Mortality Surveillance, based on a missed opportunity, 65.5% were considered avoidable, of which 77.0% were pregnant woman with at least one antenatal care appointment. Table 2 shows the causes of maternal deaths. A total of 54.2% were due to direct causes (hypertensive disorders, hemorrhages and infections, mainly); 45.8% were due to indirect causes (circulatory system diseases, digestive system diseases, pre-existing hypertension and respiratory system diseases).

**Table 2:** Causes distribution of maternal death

Death cause (ICD 10)	No.	%
Direct Obstetric causes	45	52.0
Hypertensive Disorders (O12-O16)	13	15.0
Hemorrhage (O72-O72.2)	07	8.0
Infections (O85-O86.8)	06	7.0
Embolism (O88.1)	03	3.5
Pregnancy leading to abortion (O01-O08) Premature Placental Separation (O45-O45.9) Genitourinary system infection (O23.1-O23.4) Circulatory system disease (O99.4) Puerperal cardiomyopathy (O90.3) Respiratory system disease (O99.5) Excessive vomit during pregnancy (O21.1) Other kinds of uterine atony (O62.2) Deep thrombophlebitis during puerperium (O87.1) Infection in the amniotic sac and membranes (O41.1)	03 02 02 02 02 01 01 01 01 01	3.5 2.5 2.5 2.5 2.5 1.0 1.0 1.0 1.0 1.0
Indirect Obstetric causes	38	43.5
Circulatory system diseases (O99.4)	09	10.5
Digestive system diseases (O99.6) Respiratory system diseases (O99.5)	04 03	5.0 3.5
Chronic systemic arterial hypertension (O10-O11) Viral hepatitis complicating pregnancy (O98.4) Genitourinary system infection (O23) Other specified diseases and affections (O99.8) Obstetric origin embolism (O88.2-O88.8) Post-labor coagulation deficiency (O72.3) Puerperal Infection (O85) Pregnancy leading to abortion (O07.8) Pregnancy complications sequelae (O94) Non-specified puerperal complication (O90.9) Other viral diseases (O98.5) Nervous system diseases (O99.3) Other kinds of uterine atony (O62.2) Assistance performed due to uterine tumor body (O34.1) Other puerperal complications (O90.8) Other non-specified urinary system infections (O23.9) Inconclusive (Cases 1, 2, and 3)* Late Maternal Death	03 02 02 02 02 01 01 01 01 01 01 01 01 01 01 01 03 01	3.5 2.5 2.5 2.5 2.5 1.0 1.0 1.0 1.0 1.0 1.0 1.0 1.0 1.0 1.0 1.0 3.5 1.0
Total	87	100.0

*Inconclusive cases: Case 1) 28 years old, single, brown, with antenatal care, cesarean section, death in the postpartum period, live newborn, gestation duration from 37 to 41 weeks, low socioeconomic status. Case 2) 19 years old, single, white, with antenatal care, cesarean section, postpartum death, live newborn, gestation duration 36 weeks, low socioeconomic status. Case 3) 30 years old, single, white, tubal pregnancy, did not give birth, middle socioeconomic status.

Table 3 shows the results of association and multiple logistic regression tests. There was a significant association between maternal death and women's age (p = 0.0008), antenatal care (p < 0.0001), number of pregnancies (p < 0.0001), and the type of delivery (p = 0.0042). All women with no antenatal care (n = 10) and with multiple pregnancies (n = 31) were from case group, so that was not possible to calculate their odds ratio. The results of multiple logistic regression analysis showed that older pregnant women (OR = 2.63) or those with cesarean delivery (OR = 2.51) were more prone to maternal death.

**Table 3:** Factors associated to increased risk of maternal death, by association and multiple logistic regression analysis for maternal death

Variable	N (% ^$^ )	Maternal Deaths – Cases (n = 87)	Non-deaths – Controls (n = 348)	^&^ crude OR ( ^#^ IC95%)	p-value	^&^ OR adjusted( ^#^ IC95%)	p-value
		N (% ^$^ )					
Age (years)							
< 35	367 (84.4)	63 (72.4)	304 (87.4)	Ref		Ref	
≥ 35	68 (15.6)	24 (27.6)	44 (12.6)	2.63 (1.49-4.64)	0.0008	2.63 (1.41-4.91)	0.0025
Education (years)							
< 4	11 (2.8)	2 (3.8)	9 (2.6)	1.47 (0.31-7.01)	0.6258	−	−
≥ 4	389 (97.2)	51 (96.2)	338 (97.4)	Ref			
Marital status							
Single / Divorced / Widow	184 (42.5)	41 (48.2)	143 (41.1)	1.34 (0.83-2.15)	0.2332	−	−
Married	249 (57.5)	44 (51.8)	205 (58.9)	Ref			
Antenatal care							
Yes	416 (97.6)	70 (87.5)	346 (100.0)		<0.0001	−	−
No	10 (2.4)	10 (12.5)	0 (0.0)	−			
Number of Appointments							
0-3 appointments	81 (20.1)	10 (17.2)	71 (20.6)	0.80 (0.39-1.67)	0.5579	−	−
> 4 appointments	322 (79.9)	48 (82.8)	274 (79.4)	Ref			
Gemelarity							
No	396 (97.3)	56 (94.9)	340 (97.7)	Ref		−	−
Yes	11 (2.7)	3 (5.1)	8 (2.3)	2.28 (0.59-8.84)	0.2346		
Pregnancy							
Nulliparous ^∼^	31 (7.3)	31 (38.8)	0 (0.0)	−	<0.0001	−	−
Multiparous	393 (92.7)	49 (61.3)	344 (100.0)				
Type of delivery							
Vaginal	144 (34.6)	13 (19.1)	131 (37.6)	Ref		Ref	
Cesarean	272 (65.4)	55 (80.9)	217 (62.4)	2.55 (1.34-4.85)	0.0042	2.51 (1.32-4.80)	0.0053
Socioeconomic							
Low	203 (46.8)	46 (53.5)	157 (45.1)	1.53 (0.83-2.83)	0.1722	−	−
Medium	125 (28.8)	23 (26.7)	102 (29.3)	1.18 (0.59-2.35)	0.6368		
High	106 (24.4)	17 (19.8)	89 (25.6)	Ref			

$Percentage in the column; &Odds ratio; #Confidence Interval; ∼Among women in their first pregnancy, 45% showed comorbidities (50% hypertensive disorders), 84% attended antenatal appointments, 55% had more than 4 appointments, 6% had twin pregnancies, 10% aged 35 years or older and 61% underwent cesarean deliveries. Statistical tests: simple and multiple logistic regression models.

**Fig. 1:**
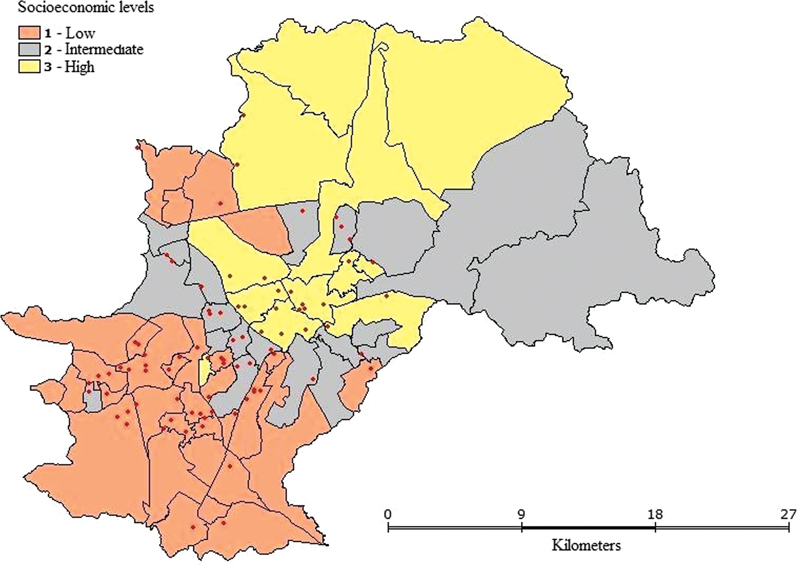
Spatial distribution of maternal deaths.

The spatial distribution showed that 53.5% of deaths occurred in the low socioeconomic level (1), 26.7% in the intermediate level (2) and 19.8% in the high level (3). Deaths were dispersed throughout the urban territory and no formation of cluster was observed ( Figure 1 ). Only a minor concentration of maternal deaths occurred in low-socioeconomic level stratum, or the most vulnerable areas of the city. However, this discrete concentration was not enough to characterize a cluster.

Maternal Mortality Ratio (MMR) for the period 2000-2015 was 37.1 death per 100.000 live births (37.1/100,000LB).

## Discussion

This study assessed maternal deaths and related factors in a Brazilian large-sized municipality during 2000-2015. Maternal mortality is a serious public health issue and became a social development indicator and it is considered an individual, family and social tragedy. 20

Our findings showed that women aged 35 years or more were more prone to death than younger women, result similar to others. 9 17 Age has been considered an important factor to assess the pregnancy's risk, since women older than 35 years can be more prone to preeclampsia. 21 Maternal age was significantly associated to preeclampsia in a study conducted in Sweden and China. 21 These findings imply that attention should be given to older pregnant women in order to diagnose early the risks of pregnancy-induced hypertension with proper monitoring of clinical status. For preeclampsia prevention, only calcium supplementation (calcium carbonate, 1,000–2,000 mg/day) and low-doses aspirin daily (50–170 mg) are considered effective in clinical practice. 22

Cesarean delivery prevalence in the present study was 65.4%, a superior rate than that found (43.3%) among Brazilian pregnant women assisted at the Unified Health System (SUS). 23 Pregnant women with cesarean delivery presented more chance of dying than those with vaginal birth. Studies show that cesarean delivery can contribute to increase the risk of maternal death 17 24 and maternal morbidity. 25 Cesarean delivery should be indicated properly and corresponds to a strategy of future pregnancy-related deaths prevention when only performed when medically indicated. 9 This study did not investigate the presence of obstetric morbidity and the proper cesarean indication.

Antenatal care was highly associated with maternal death in our study. In a similar study an inverse and significant correlation between maternal mortality ratio and antenatal care coverage was found. 11 The proper antenatal care classifies the pregnant woman's risk and specialized professionals can monitor it. 26 Although the municipality has a pregnancy risk classification protocol, 27 among the avoidable maternal deaths, 77% of pregnant women attended antenatal care, a fact that suggests a need for investigating the quality of the antenatal care and hospital assistances.

Number of previous pregnancies presented a highly significant association with maternal mortality. First pregnancy showed more chances of maternal death in comparison with the multiparous ones. Similar results were found in China and Sweden where being nulliparae was highly associated with preeclampsia. 21 By analyzing our data bank, among women in their first pregnancy, 45% showed comorbidities (50% hypertensive disorders), 84% attended antenatal appointments, 55% had more than 4 appointments, 6% had twin pregnancies, 10% aged 35 years or older and 61% underwent cesarean deliveries. The fact that almost half of women showed comorbidities could partially explain our findings, since such pregnant women should have a proper risk classification and monitoring. Recent study in the United States shows that pregnant women are presenting poorer health overtime. 28

The MMR was 37.1/100,000LB, a value within Brazilian findings that ranged from 29.4/100,000LB to 83.3/100,000LB. 27 Recent data showed a Brazilian MMR of 57.6/100,000 live births 29 and 60/100,000LB, 1 whereas a global MMR of 211/100,000LB. 1 Projection of MMR for 2030 based on the Sustainable Development (SDG) goal is less than 70/100,000LB for the world and that none country should have a MMR over 140/100,000LB. Therefore, efforts at all government levels should be directed towards this goal.

According to spatial analysis, although no formation of cluster was observed, a discrete concentration of deaths in the low socioeconomic level was verified. Therefore, a need for continuous investments in public policies aimed to ensure access equity and quality in health care is suggested.

In the present study, 54% of maternal deaths were due to direct causes such as hypertension, hemorrhage and infections, corroborating with other Brazilian study 29 and similar to data from maternal death worldwide. 30 Direct obstetric causes result from obstetric complications during pregnancy, labor or puerperium, whereas indirect causes result from pre-existing medical conditions that were aggravated by pregnancy. 1 The findings of this study and of two previous investigations in the same municipality indicate a decrease in the prevalence of direct causes. 31 32 However, 61% of the deaths by direct causes were avoidable, indicating the need for improving access and quality in the antenatal, labor and providing puerperium assistance, and for providing efficient actions for the reduction of maternal mortality. Causes of maternal mortality are related to the development level of a region. Less developed ones usually have more deaths due to direct causes such as hypertension diseases, hemorrhage and infections. Such causes tend to decrease progressively as the region develops so that indirect causes usually related to more complex diseases prevail. 33

An important action that could be efficient in the prevention of maternal mortality is the incorporation of maternal near miss analysis by assessing cases of pregnant women with serious complication during antenatal, labor or puerperium and that survived. The study of such cases can subside healthcare systems to deal with maternal near miss in a multidimensional manner. 34

Maternal Mortality rate can also be affected during times of crisis. In a recent ecologic study performed in Brazil, the authors evaluated the impact of COVID-19 pandemic on maternal mortality by comparison of data from 2020 with data from 2010-2019 and found an increase of 40% on maternal mortalities on pregnancies considered low-risk. 35 Authors of a multinational cohort study, found similar results, showing increase in severe morbidity and mortality. 36 During health emergencies specific programs should be proposed and implemented to protect the health of the pregnant women and their offspring.

As a limitation of this study, we should mention the time frame of more than 5 years, the use of secondary data and the absence of external validity of the results. The strengths include the quality of data since all maternal deaths were investigated by the Municipal Committee of Surveillance in Maternal and Child Mortality.

## Conclusion

In conclusion, maternal deaths were mostly avoidable, distributed dispersedly among the different socioeconomic levels and more prone to occur among older women or those undergoing cesarean deliveries. The development of programs to increase awareness of the risks of cesarean section and risks associated to advanced maternal age together with improvement on quality of care may have an impact on the maternal mortality.
